# IMPACT OF MELD SODIUM ON LIVER TRANSPLANTATION WAITING LIST

**DOI:** 10.1590/0102-672020190001e1460

**Published:** 2019-12-09

**Authors:** Alexandre Coutinho Teixeira de FREITAS, Aline Tatiane RAMPIM, Carolline Popovicz NUNES, Júlio Cezar Uili COELHO

**Affiliations:** 1 Department of Surgery, Federal University of Paraná, Curitiba, PR, Brazil; 2 Medical School, Federal University of Paraná, Curitiba, PR, Brazil

**Keywords:** Liver transplantation, Waiting list, Hyponatremia, Mortality, Transplante de fígado, Lista de espera, Hiponatremia, Mortalidade

## Abstract

**Background:**

Serum sodium was incorporated to MELD score for the allocation of liver transplantation In the USA in 2016. Hyponatremia significantly increased the efficacy of the score to predict mortality on the waiting list. Such modification was not adopted in Brazil.

**Aim:**

To carry out a simulation using MELD-Na as waiting list ordering criteria in the state of Paraná and to compare to the list ordered according to MELD score.

**Methods:**

The study used data of 122 patients waiting for hepatic transplantation and listed at Parana´s Transplantation Central. Two classificatory lists were set up, one with MELD, the current qualifying criteria, and another with MELD-Na. We analyzed the changes on classification comparing these two lists.

**Results:**

Among all patients, 95.1% of the participants changed position, 30.3% showed improvement, 64.8% presented worsening and 4.9% maintained their position. There were 19 patients with hyponatremia, of whom 94.7% presented a change of position, and in all of them there was an improvement of position. One hundred and one patients presented sodium within the normal range and 95% of them presented a change of position: Improved placement was observed in 18.8%, and worsened placement in 76.2%. Two patients presented hypernatremia and changed their position, both worsening the placement. There was a significant different behavior on waiting list according to sodium serum level when MELD-Na was applied.

**Conclusion:**

The inclusion of serum sodium caused a great impact in the classification, bringing benefit to patients with hyponatremia.

## RESUMO

**Racional:**

Desde 2016 os EUA utilizam o MELD-Na para alocação de enxertos hepáticos, uma vez que o valor da natremia, quando adicionada ao MELD, aumenta a eficácia para prever a mortalidade na lista de espera. Entretanto, tal modificação não foi adotada no Brasil.

**Objetivo:**

Realizar uma simulação utilizando o MELD-Na como critério de ordenamento na lista de espera para transplante hepático no estado do Paraná.

**Métodos:**

O estudo utilizou os dados cadastrais de 122 inscritos na lista de espera para transplante hepático da Central Estadual de Transplantes do Paraná. Duas listas classificatórias foram montadas, uma utilizando o MELD e outra o MELD-Na. Foram analisadas as alterações na ordem classificatória dos pacientes comparando essas duas listas.

**Resultados:**

Entre todos os pacientes, 95,1% mudaram de posição: 30,3% apresentaram melhora, 64,8% apresentaram piora e 4,9% mantiveram a sua posição. Dos 19 pacientes com hiponatremia, 94,7% apresentaram mudança, todos para melhor posição. Dos 101 pacientes com sódio normal 95% apresentaram mudança de posição: em 18,8% houve melhora e em 76,2% houve piora da colocação. Dois pacientes apresentaram hipernatremia e ambos mudaram de posição para pior colocação. Houve diferença significativa entre os diferentes níveis de sódio em relação ao comportamento na lista de espera quando aplicado o MELD-Na.

**Conclusão:**

A inclusão do sódio sérico causou grande impacto na classificação, trazendo melhora na posição dos pacientes com hiponatremia.

## INTRODUCTION

Liver transplantation was first performed in the early 1960s by Thomaz Starzl in the United States of America. In a few decades it has become the procedure of choice for treatment of patients with end-stage liver cirrhosis^18^. In Brazil, the first transplant was carried out in 1968 at the Hospital das Clínicas, Medical School, University of São Paulo^7^. Since then, the number of transplants performed in the country has significantly increased^8^ ^,^ ^12^. Thus, it was necessary to create criteria to organize the list of patients waiting for an organ. In the 1990s, the transplantation community in Brazil adopted the “single list” for all enrolled patients^12^. This list generated great questions, because it used the chronological order for distribution of organs. Modifications have been discussed over the years, always aimed at ensuring fairness in the distribution of organs^12^. After all, although liver transplants save lives, organs are a scarce resource.

In 2006, the Ministry of Health in Brazil published the Decree n^0^. 1160, which modified the criteria for distribution of liver from cadaveric donors for transplantation, establishing the implantation of the MELD (Model for End-stage Liver Disease) system for adult recipients and the Pediatric End-Stage Liver Disease (PELD) system for pediatric recipients^13^. This is a mathematical model that estimates the mortality risk of a patient with terminal liver disease based on the following laboratory tests: total bilirubin, creatinine and INR^13^. This model was previously developed to predict the survival of cirrhosis patients subjected to transjugular intrahepatic portasystemic shunt (TIPS), since some studies have proven that it could be used as a reliable tool to evaluate survival in patients with chronic liver disease^10^ ^,^ ^14^. In general, MELD presents two important advantages in the search for better hepatic allocation. First, it uses only objective variables that are patient-specific and do not require observer interpretation. Second, it estimates the risk of mortality, an important parameter to define the need for transplant^15^ ^,^ ^21^.

Despite the usefulness of MELD, studies have shown that this score may not accurately reflect the risk of death in some groups of patients, such as those with hyponatremia^9^, an important predictor of mortality in patients listed for liver transplantation^10^. It is a frequent event in cirrhotic with ascites and is considered an independent predictor of long-term survival in hepatorenal syndromes^2^. Therefore, in order to increase the effectiveness, it was proposed the inclusion of serum sodium level in the calculation of MELD (MELD-Na)^3^. This change was adopted by the United States in January 2016 for allocation of hepatic grafts^19^. In Brazil, however, this change has not yet been implemented, continuing to use MELD as a qualifying criterion^13^.

The objective of this study was to perform a simulation using the MELD-Na as waiting list ordering criteria for liver transplantation in the state of Paraná, Brazil, and thus evaluate the impact on the classification of patients compared to the ordered list according to the MELD.

## METHODS

The study was approved by the Research Ethics Committee of the Health Sciences Sector of the Federal University of Paraná, under the number 2,199,554, and by the Ethics and Research Committee of the State Department of Health of Paraná, under the number 2,243,844.

It was used data of patients waiting for liver transplantation at the State Center of Transplantation of Paraná. The information of interest for the research was collected in a standardized way in the registries of this state organ. The information analyzed was: blood type; age; gender; etiology of cirrhosis; date of entry on waiting list; total bilirubin; INR; creatinine and serum sodium. Data collection was carried out on September 30^th^, 2017, through the Computerized Management System, of which access was permitted by the State Transplant Center of Paraná. It was not necessary to collect any additional data that was not already available in the computer system. Patients were not identified by name but by waiting list position defined by MELD score for each blood type. 

MELD calculation was performed using the formula “MELD=10* (0.957* ln [Creatinine]) + (0.378* ln [Bilirubin]) + (1.12 ln [INR])) + 6.43”. MELD-Na was calculated through the formula “MELD-Na=MELD + 1.32 x (137 - Na) - [0.033 x MELD*(137 - Na)]”. Serum sodium value was corrected for the range of 125-137 mEq/l, according to criteria determined by UNOS (United Network for Organ Sharing). Based on MELD and MELD-Na values, two different classification lists were set up for each blood type (A, B, AB and O). In case of a tie, waiting time in the list was used as the tiebreaker criteria. Based on these two lists, the patient’s position in the classification list was analyzed using MELD-Na compared to the position in the classification list using MELD. In this evaluation it was recorded whether the patient presented a change in position or not and, in case of change, whether it was an improvement of the position or a worsening. Changing the waiting list position in each blood type was also assessed by dividing the patients into three groups according to serum sodium level: hyponatremia (Na<135 mEq/l), normonatremia (Na≥135 mEq/l≤145 mEq/l) and hypernatremia (Na>145 mEq/l).

### Statistical analysis

Associations between groups were characterized using inferential statistics through the cross-table independence test and the Fisher›s test. The confidence interval (CI) analysis was performed using the T-Test. Paired sample analysis was performed using the Wilcoxon test. The correlations between the criteria and the changes of positions were evaluated by Spearman’s correlation test. The level of statistical significance was set at 5%. Statistical analysis was performed using the program Action Stat Version 3.4.124.1308 build 3.

## RESULTS

A total of 122 patients were included in the study. Clinical characteristics and demographic data are shown in Table 1. The mean age was 51.6 years (ranging from 18 to 68 years). Of the 122 patients, 81 were male and 41 female. Regarding blood type, 62 patients were type A, 9 type B and 51 type O. There were no patients with blood type AB. The average waiting time for the waiting list was 185 days (ranging from 1 to 1647 days). The main causes of liver diseases were alcohol (35%), viral hepatitis (21%), cryptogenic (16%), and fatty liver disease (6%). 

**TABLE 1:** Patients clinical characteristics and demographic data

	n/Mean	Standard deviation/%
Patients	122	
Age (years)	51,6	10,38
Male/Female	81/41	66,4%/33,6%
Cause of disease		
Alcoholic	43	35,25%
Viral hepatitis	26	21,31%
Cryptogenic	20	16,4%
Hepatic steatosis	8	6,56%
Autoimmune	6	4,92%
Others	19	15,58%
Blood type		
A	62	
B	9	
O	51	
MELD	15,09	2,64
MELD-Na	16,01	3,06
Serum sodium	137,88	4,02
Serum creatinine	0,92	0,3
Total bilirubin l	2,96	3,15
INR	1,5	0,26

The mean serum creatinine was 0.92 mg/dl, the mean total bilirubin was 2.96 mg/dl and the mean INR was 1.5. Serum sodium had an average value of 137.88 mEq/l (ranging from 121 to 146).

**FIGURE 1:**
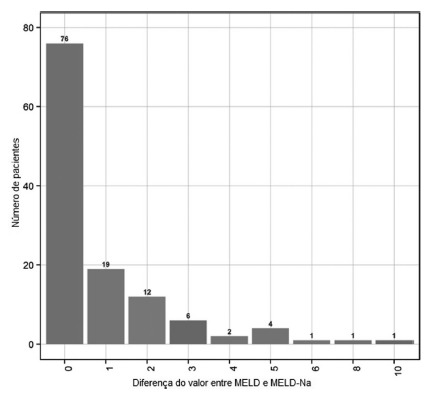
Differences between the value of MELD and MELD-Na

The mean MELD value was 15.09, and the MELD-Na was 16.01. The difference between the MELD and MELD-Na values are shown in Figure 1, and the mean variation was 0.93. Among patients with hyponatremia (Na<135 mEq/l), the mean variation was 4.1 (ranging from 2 to 10).

**FIGURE 2:**
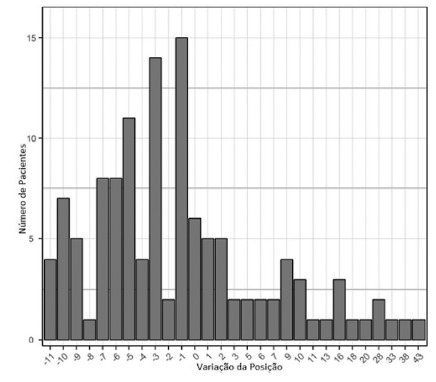
Variation of position in the waiting list according to MELD-Na in relation to MELD

General evaluation of all 122 patients in whom MELD-Na was applied showed six (4.9%) remaining in the same position on the waiting list, 79 (64.8%) presented worse positions and 37 (30.3%) better positions (Figure 2). It was observed a statistical difference between the two lists (p=0.02).

In patients with blood type A, 30.60% improved their waiting list position, with an average variation of 12.42 positions, the largest being a variation of 38 positions. In addition, 69.4% worsened their position, mean variation of 5.49 positions, the largest being a variation of 11 positions. In patients with blood type B, 33.33% improved their position, with an average variation of three positions; 66.37% worsened their position, with an average variation of 1.5 positions. In patients with blood type O, 33.33% improved their position, with average variation of 9.64 positions, being the greatest variation of 43 positions; 66.37% worsened their position, with an average change of 4.82 positions.

In 62.3% of patients no difference was observed between absolute values of MELD and MELD-Na. Absolute score value presented alteration in 37.7%. Among those, 80.4% had improvement in position, 2.2% maintained the position and 17.4% presented worsening in position. For those who presented improvement in position the average change was 8.4 positions, and there was a gain of 2.46 positions for each point added using MELD-Na. Observing the lowest MELD score half population, there was an improvement of 11.26 positions, in which the gain was 3.42 positions for every one point added to MELD by the introduction of serum sodium in the formula. In the half with the highest MELD scores, the mean variation was 5.56 positive positions, with gain of 1.25 positions for each point gained with the MELD-Na. There was a significant correlation between MELD and position variation and also between MELD and the position/point variation ratio (p<0.01).

**FIGURE 3:**
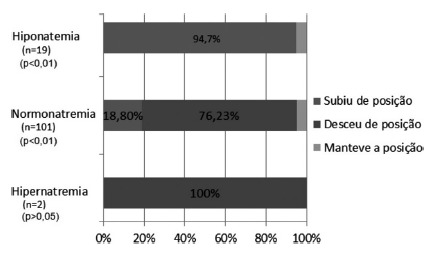
Waiting list change of position according to serum sodium level

Figure 3 demonstrates the changes in the waiting list according to serum sodium stratification. There was a significant difference between the different levels of sodium and the behavior on the waiting list when applied MELD-Na (p<0.01). There were a total of 19 patients with hyponatremia (<135 mEq/l), representing 15.6% of the total. Of these, 94.7% presented a change of position (p<0.01), and in all cases there was an improvement of position, with a mean variation of 16.42 positions (95% CI [10.4, 22.4]). One hundred and one patients had sodium within the normal range (135-145 mEq/l), representing 82.8% of the total. Of these, 95% presented a change of position: in 18.8% there was improvement of the placement (p<0.01), with a mean variation of 4.48 positions (95% CI [3, 6.5]), and in 76, 23% had worsening of the placement (p<0.01), with an average variation of 5.15 positions (95% CI [-5.9, -4.4]). Two presented hypernatremia (>145 mEq/l), representing 1.6% of the total. Of these, 100% changed position (p>0.05), and in 100% there was worsening of the placement, with a mean change of three positions (95% CI [-22.4, 28.4]).

## DISCUSSION

Organs for transplantation are regarded as a limited resource. Currently, there is a constant pursuit for criteria that ensure the best form for its distribution. Several studies have shown that the serum sodium, when incorporated into the MELD calculation, significantly increased the efficacy of the score to predict liver transplant waiting list mortality^4^ ^,^ ^6^ ^,^ ^16^. A study of 6769 patients showed that 7% of deaths on the waiting list could be avoided if MELD-Na had been used instead of MELD^11^. Based on several studies, UNOS (United Network for Organ Sharing) approved the incorporation of serum sodium in the calculation of the MELD score for allocation of liver transplantation. This was implemented in January 2016 in the United States of the America^19^. The model defined by the Scientific Registry of Transplant Recipients (SRTR), with a lower sodium limit of 125 mEq/l and an upper limit of 137 mEq/l, predicted that the use of MELD-Na for allocation would result in 52 fewer waiting list deaths per year^5^. In Brazil this change has not been implemented yet and MELD remains the classification criteria^13^.

This study evaluated the impact of adopting serum sodium on MELD to classify patients on liver transplant waiting list. Until the moment of publication this is the first study in Brazil to analyze this issue and focusing on position variation and behavior on the list. We found that 94.7% of patients with hyponatremia had a position change. All of them improved their position. A patient with hyponatremia would be benefited with the adoption of MELD-Na as organ allocation criteria leading to an anticipation of the procedure. One study that incorporated serum sodium to MELD showed that it would affect 27% of transplant recipients^4^.

The benefit to patients with low serum sodium is important, since hyponatremia is associated with cirrhosis complications such as refractory ascites and hepatorenal syndrome^1^. A prospective study conducted by Borroni et al.^6^ showed that mortality was significantly higher in cirrhotic patients with hyponatremia compared to those without this complication (26 vs. 9%). Moreover, in a study conducted by Kim et al.^11^ performed with patients waiting for liver transplantation a 5% risk of death increase was observed for each decrease of one sodium unit within the range of 125 to 140 mEq/l.

The relationship between serum sodium value and list position was assessed by categorizing patients according to natremia (high, normal or reduced serum sodium). This analysis showed that the impact was greater in patients with hyponatremia, leading to a gain of 16.42 positions on the list in average. Patients with sodium values within the normal range and also those with hypernatremia had a negative variation of approximately three positions. These data are in agreement with the literature which describes that the adoption of MELD-Na resulted in little or no increase in the score for patients with normal serum sodium levels, but that patients with low serum sodium levels were benefited, receiving a much higher priority score, corresponding to their mortality risk^4^. Analyzing the data, we conclude that there is a significant dependence between the different levels of sodium in the blood compared to the behavior on the waiting list applying MELD-Na, wherein there is a direct correlation between hyponatremia and improved position in the list.

The mean MELD score found was 15.09 and mean serum sodium value was 137.88 mEq/l, which were very close to the values found in a study conducted by Kim et al em 2008^11^, who found the MELD score of 15 and sodium value of 137 mEq/l. Hyponatremia (Na<135 mEq/l) occurred in 15.6% of the total patients. This is within the range of values described in the literature (8% to 31)^4^ ^,^ ^11^. In addition, it was observed that the majority of patients had sodium values higher than 137 mEq/l, and in these cases the value of MELD-Na was identical to that of MELD. Such situation was expected since the formula used to calculate MELD-Na is not intended to harm patients with serum sodium above this value, and higher serum sodium values are adjusted to 137 mEq/l which results in a MELD identical to MELD-Na.

Only 37.7% of patients presented a change in score comparing MELD and MELD-Na. This situation was predicted by Biggins et al.^4^, who stated that since the MELD-Na score differs substantially from the MELD score only for patients with hyponatremia, the proportion of candidates for liver transplantation who would be affected by the use of this combined score would be modest. In fact, the proportion of transplant candidates affected by the adoption of serum sodium in the calculation is small. But in these patients the magnitude of the difference between MELD-Na and MELD is large enough to make a real difference in the probability to receive an organ. For example, in this study one of the participants ranged from 38 positions, going from 39^th^ place to 1^st^ place on the list of blood type A. This significant variation was due to the increase of the MELD from 15 to MELD-Na of 25. This is relevant because despite not modifying the score value for all patients, MELD-Na has a better predictive death risk value in patients who are more ill^4^ ^,^ ^17^ .

Moreover, it was observed that among patients who had score difference with the inclusion of serum sodium, those with higher MELD had a smaller impact on position variation. The half population with lowest initial MELD gained 3.42 positions for every one point of MELD score increased with adoption of MELD-Na. The half with the largest MELD score, each gain of one point represented 1.25 positions. Similarly, Biggins et al^3^ found that for patients with higher MELD, the effect of hyponatremia was minimal. The effect of hyponatremia gradually decreases as the MELD score increases. However, for patients with MELD of intermediate and low values, the effect of sodium could be substantial.

The adoption of MELD-Na, prioritizing and transplanting the most severe patients earlier, also has an important financial impact. Several studies have shown that increasing the severity of liver disease predisposes to a high number of hospitalizations and high-cost procedures before transplantation^18^ ^,^ ^20^.

The design of this study was observational and cross-sectional. Data collection was done in a single day and may have resulted in patient selection bias. However, this design simulates a real situation with a new waiting list selection criteria. It is important to emphasize that external factors, such as the use of diuretics or hypotonic solutions, can modify serum sodium and consequently interfere improperly in the result of MELD-Na. However, other laboratory tests used for MELD calculation can also be affected by external factors^6^. In this study, only one way of MELD-Na calculation was evaluated. There are other formulas that incorporate sodium into MELD^6^. The objective of this study was to evaluate the impact of the same criteria currently used in the USA and that has been shown to be effective in reducing mortality risk^5^.

We suggest new studies to analyze the impact of MELD-Na on liver transplantation waiting list in Brazil. They could evaluate other ways to incorporate serum sodium into MELD calculation, waiting list time and mortality. The goal is to find the best liver allocation criteria for our country and reduce waiting list mortality.

## CONCLUSION

The incorporation of serum sodium into MELD score for liver allocation purposes causes a great waiting list impact, improving the position of patients with hyponatremia. There is a significant dependence between different levels of serum sodium and waiting list behavior, especially a direct correlation between hyponatremia and waiting list position improvement.
